# Corrigendum to ‘microRNA-21a-5p/PDCD4 axis regulates mesenchymal stem cell-induced neuroprotection in acute glaucoma’

**DOI:** 10.1093/jmcb/mjad028

**Published:** 2023-11-13

**Authors:** Wenru Su, Zuohong Li, Yu Jia, Yingting Zhu, Wenjia Cai, Peixing Wan, Yingying Zhang, Song Guo Zheng, Yehong Zhuo

**Affiliations:** State Key Laboratory of Ophthalmology, Zhongshan Ophthalmic Center, Sun Yat-sen University, Guangzhou 510060, China; State Key Laboratory of Ophthalmology, Zhongshan Ophthalmic Center, Sun Yat-sen University, Guangzhou 510060, China; State Key Laboratory of Ophthalmology, Zhongshan Ophthalmic Center, Sun Yat-sen University, Guangzhou 510060, China; State Key Laboratory of Ophthalmology, Zhongshan Ophthalmic Center, Sun Yat-sen University, Guangzhou 510060, China; State Key Laboratory of Ophthalmology, Zhongshan Ophthalmic Center, Sun Yat-sen University, Guangzhou 510060, China; State Key Laboratory of Ophthalmology, Zhongshan Ophthalmic Center, Sun Yat-sen University, Guangzhou 510060, China; State Key Laboratory of Ophthalmology, Zhongshan Ophthalmic Center, Sun Yat-sen University, Guangzhou 510060, China; Department of Clinic Immunology, The Third Affiliated Hospital at Sun Yat-sen University, Guangzhou 510630, China; Division of Rheumatology, Department of Medicine, Penn State Milton S. Hershey Medical Center, Hershey, PA 17033, USA; State Key Laboratory of Ophthalmology, Zhongshan Ophthalmic Center, Sun Yat-sen University, Guangzhou 510060, China

*Journal of Molecular Cell Biology* (2017), 9(4), 289‒301, https://doi.org/10.1093/jmcb/mjx022

In this article, there were errors in Figures 4D (western blotting analysis), 3E and F, 5G and H, and 5J and K (flow cytometry analysis).

Iba1 band in Figure 4D was the same as Cleaved caspase-3 band in Figure 2F. This was caused by carelessly mistaking the latter (Cleaved caspase-3, #1) for Iba1 band due to the high similarity between images (raw blot images of these bands from three experiments are provided in the Supplementary material). The corrected Figure 4D with updated Iba1 band (#1) and quantitation graph is shown below.

Representative FACS images for MSC and NC groups in Figures 3F and 5G, as well as that for LPS and dmiR-21 groups in Figure 5J, were extremely similar and might be derived from the same sample (FCS file). This was caused by carelessly using the same FCS file for analyzing different groups, which was not immediately discovered by authors due to the similar percentage values and shapes as expected from the two groups. All the re-exported FACS images from re-analyzing the original FCS files by Flowjo software are provided in the Supplementary material. The corrected Figures 3E and F, 5G and H, and 5J and K, with updated FACS images and quantification graphs are shown below.

These corrections do not affect the results, discussion, or conclusions. The authors sincerely apologize to the editors and readers for any inconvenience or confusion. Supplementary material is available at *Journal of Molecular Cell Biology* online.


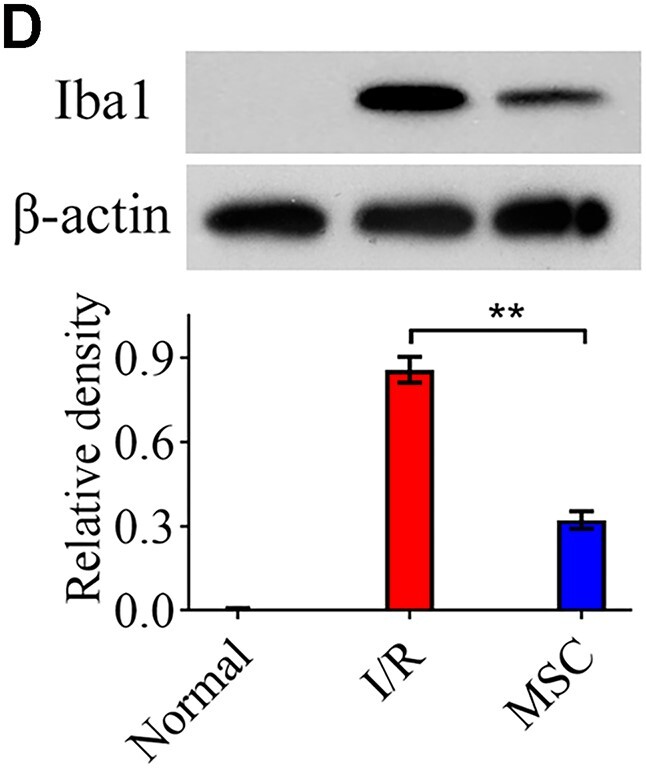


**Figure 4D**


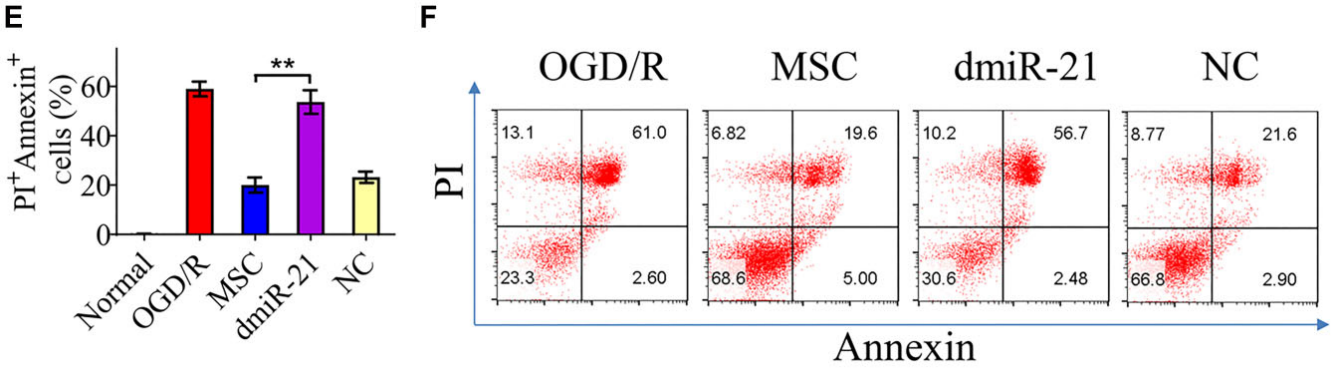


**Figure 3E and F**


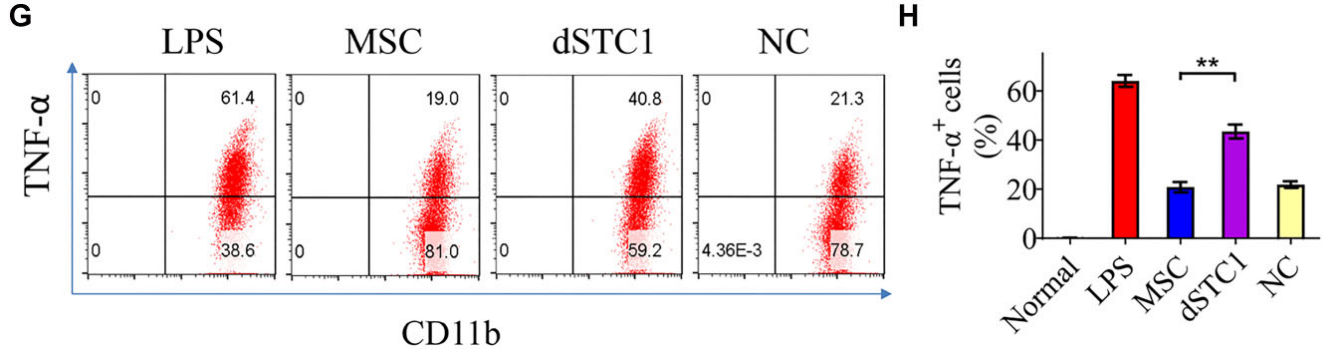


**Figure 5G and H**


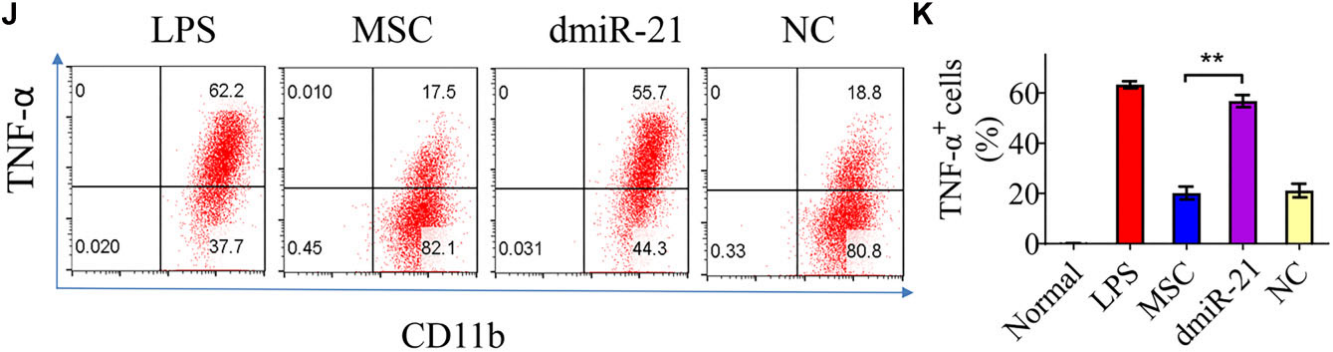


**Figure 5J and K**

## Supplementary Material

mjad028_Supplemental_File

