# Supplementary material for: Corrigendum to ‘microRNA-21a-5p/PDCD4 axis regulates mesenchymal stem cell-induced neuroprotection in acute glaucoma’
Source: J Mol Cell Biol. 2023 Nov 13;15(4):mjad028. doi: 10.1093/jmcb/mjad028 (PMC10642629; doi:10.1093/jmcb/mjad028)
Supplement: mjad028_Supplemental_File [file mjad028_supplemental_file.pdf]

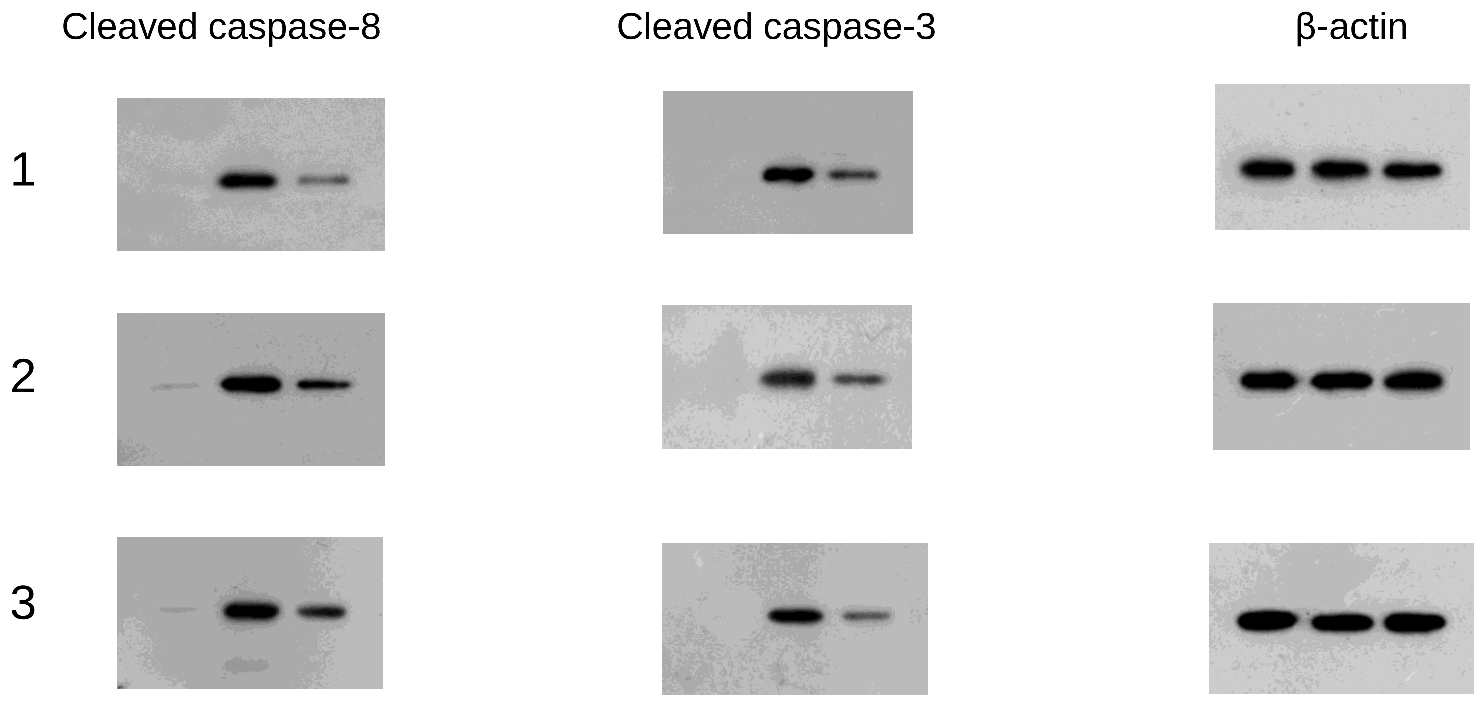

Figure 2F

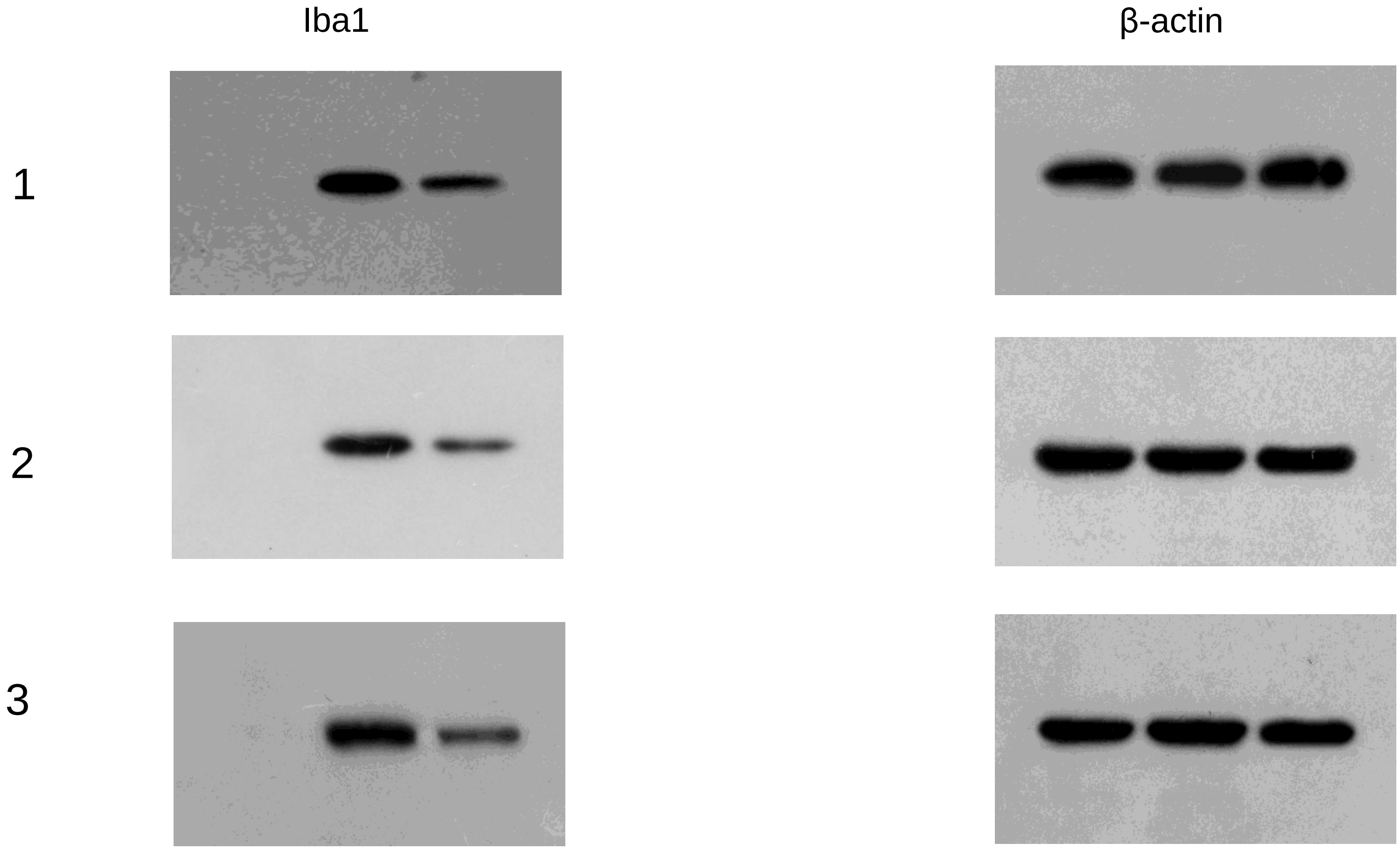

Figure 4D

Figure 2C

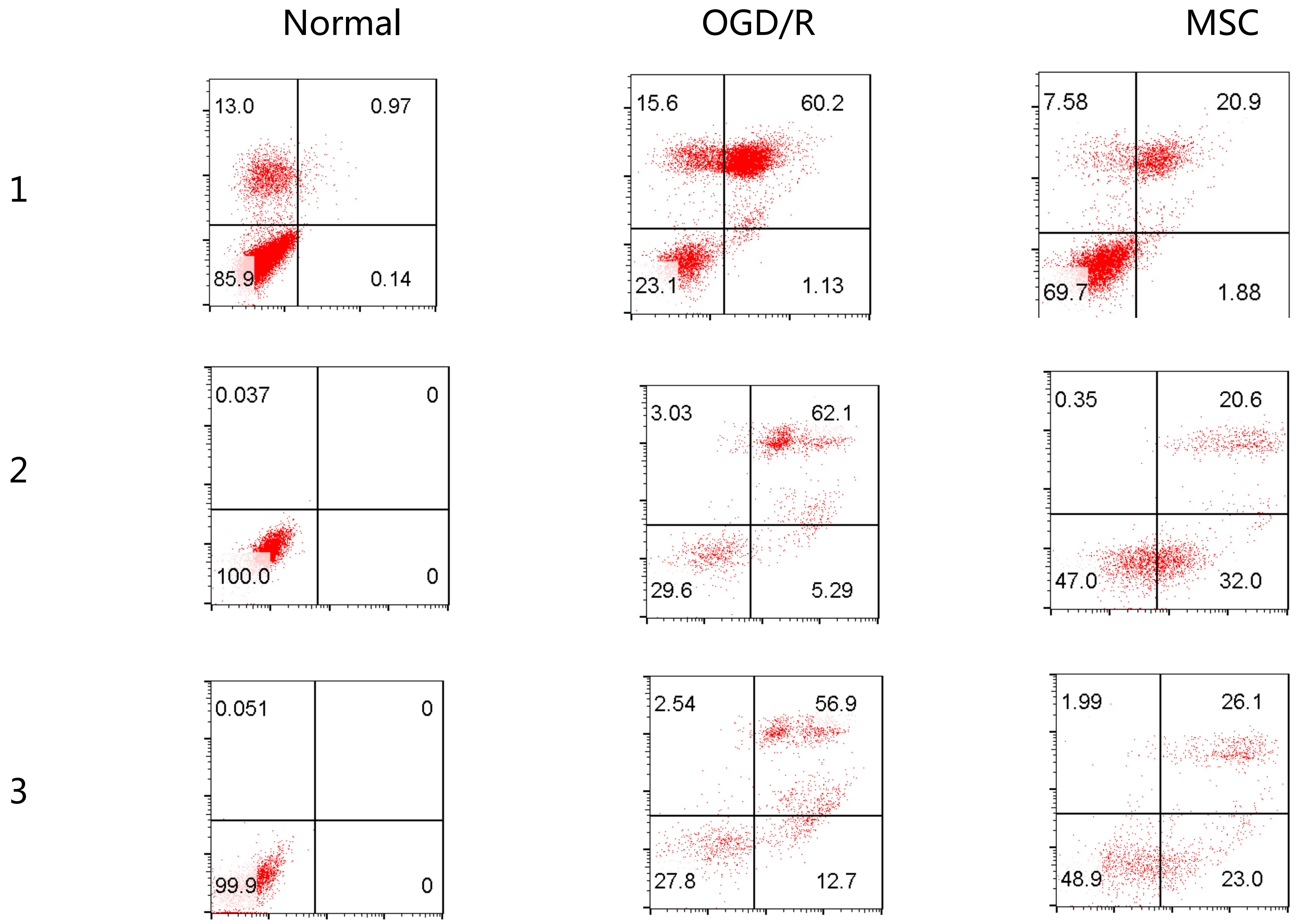
Normal
OGD/R
MSC
1
2
3
13.0
0.97
85.9
0.14
15.6
60.2
23.1
1.13
7.58
20.9
69.7
1.88
0.037
0
100.0
0
3.03
62.1
29.6
5.29
0.35
20.6
47.0
32.0
0.051
0
99.9
0
2.54
56.9
27.8
12.7
1.99
26.1
48.9
23.0

Figure 3F

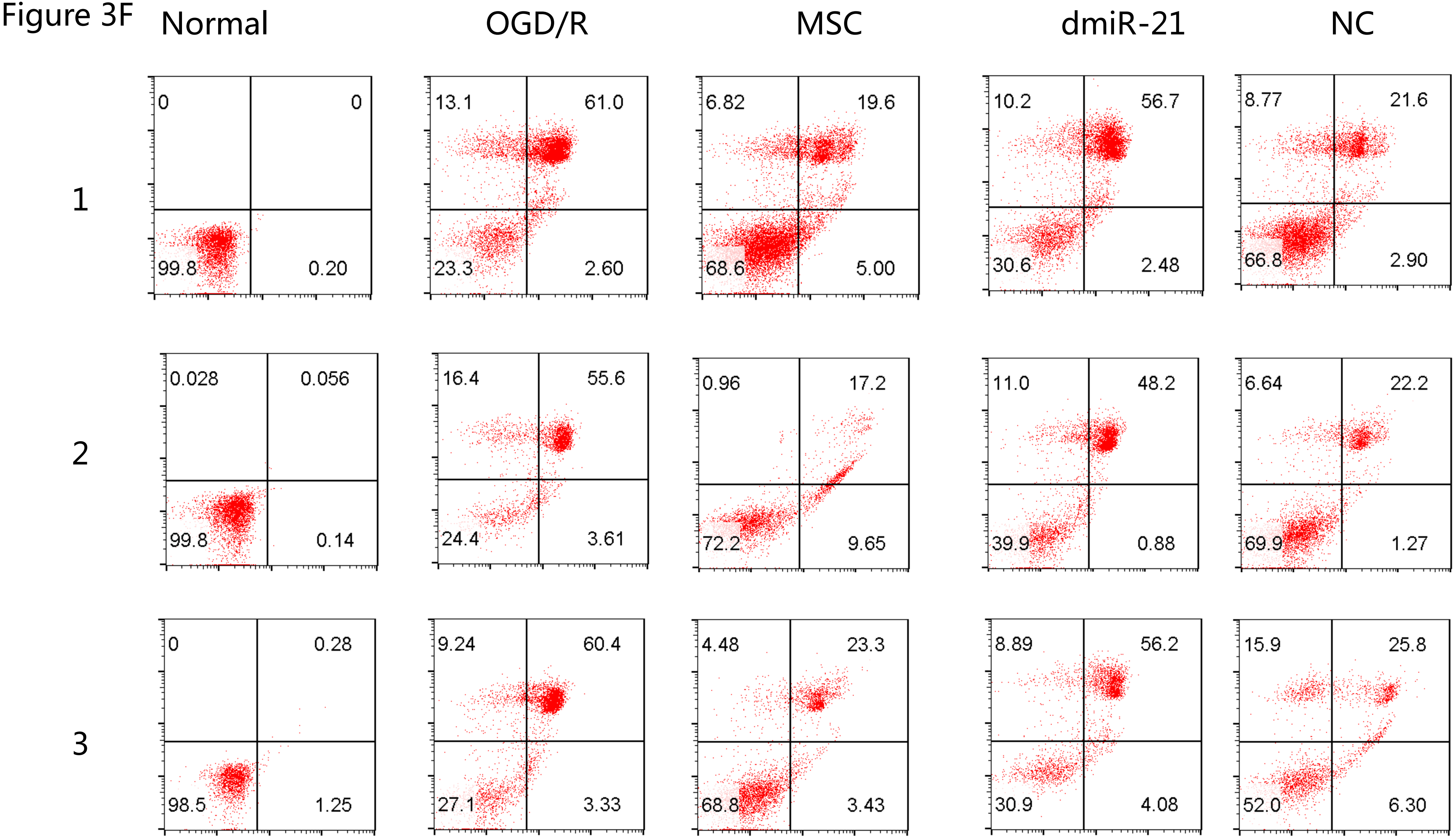

Normal
OGD/R
MSC
dmiR-21
NC
1
2
3
0
0
99.8
0.20
13.1
61.0
23.3
2.60
6.82
19.6
68.6
5.00
10.2
56.7
30.6
2.48
8.77
21.6
66.8
2.90
0.028
0.056
99.8
0.14
16.4
55.6
24.4
3.61
0.96
17.2
72.2
9.65
11.0
48.2
39.9
0.88
6.64
22.2
69.9
1.27
0
0.28
98.5
1.25
9.24
60.4
27.1
3.33
4.48
23.3
68.8
3.43
8.89
56.2
30.9
4.08
15.9
25.8
52.0
6.30

Figure 3L

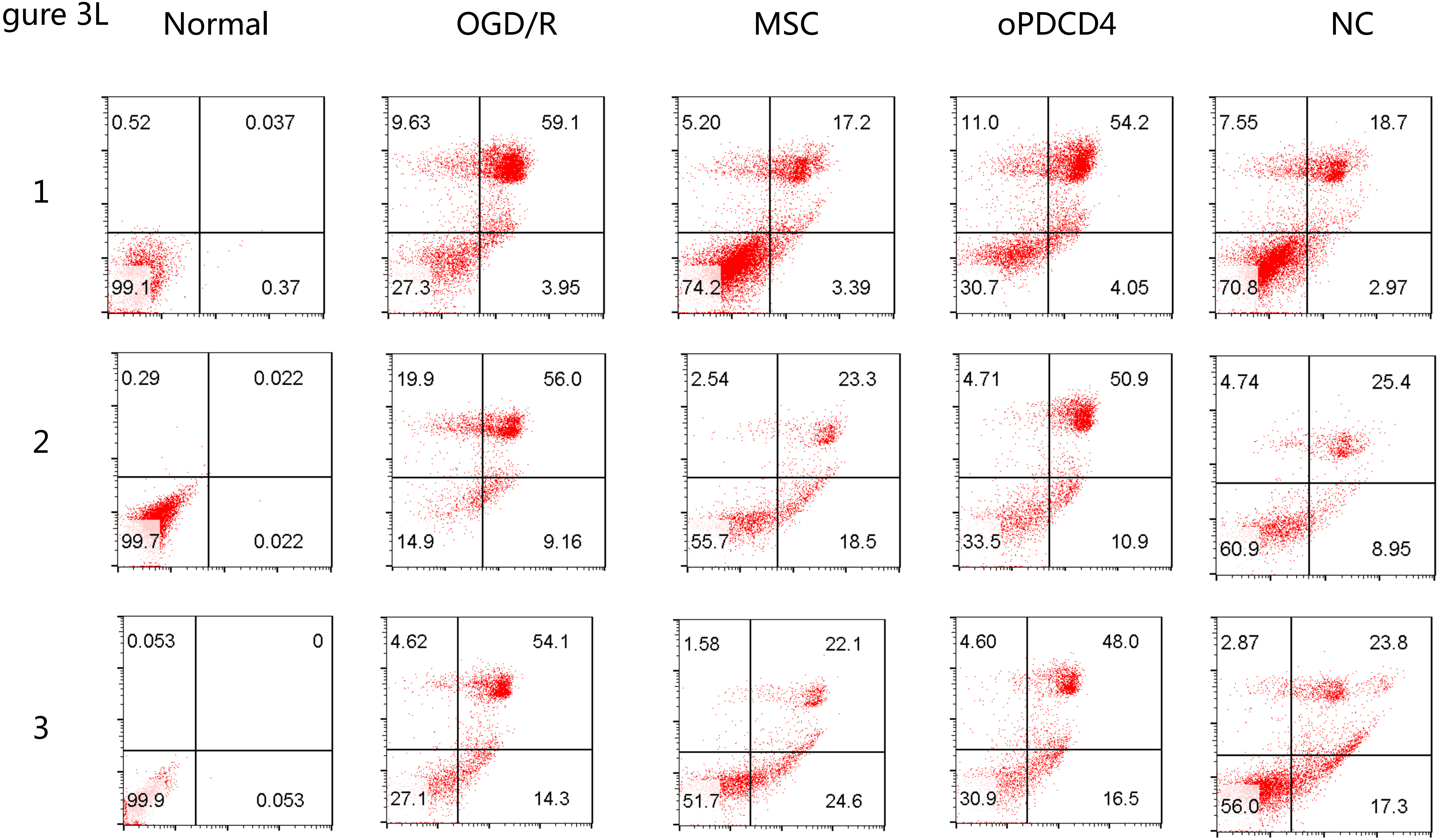
Normal
OGD/R
MSC
oPDCD4
NC
1
2
3
0.52
0.037
99.1
0.37
9.63
59.1
27.3
3.95
5.20
17.2
74.2
3.39
11.0
54.2
30.7
4.05
7.55
18.7
70.8
2.97
0.29
0.022
99.7
0.022
19.9
56.0
14.9
9.16
2.54
23.3
55.7
18.5
4.71
50.9
33.5
10.9
4.74
25.4
60.9
8.95
0.053
0
99.9
0.053
4.62
54.1
27.1
14.3
1.58
22.1
51.7
24.6
4.60
48.0
30.9
16.5
2.87
23.8
56.0
17.3

Figure 4G

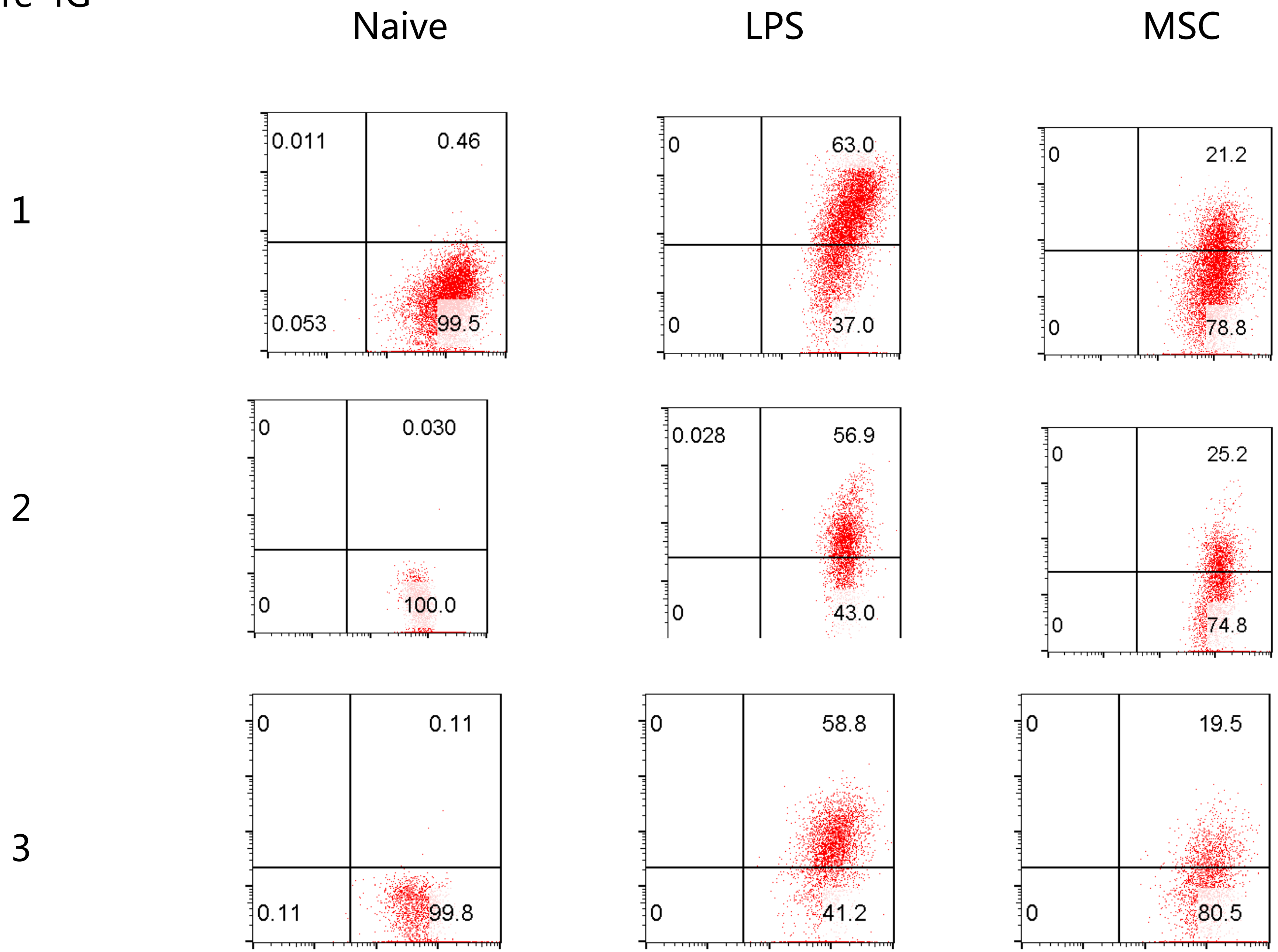
Naive
LPS
MSC
1
2
3
0.011
0.46
0.053
99.5
0
63.0
0
37.0
0
21.2
0
78.8
0
0.030
0
100.0
0.028
56.9
0
43.0
0
25.2
0
74.8
0
0.11
0.11
99.8
0
58.8
0
41.2
0
19.5
0
80.5

Figure 4I

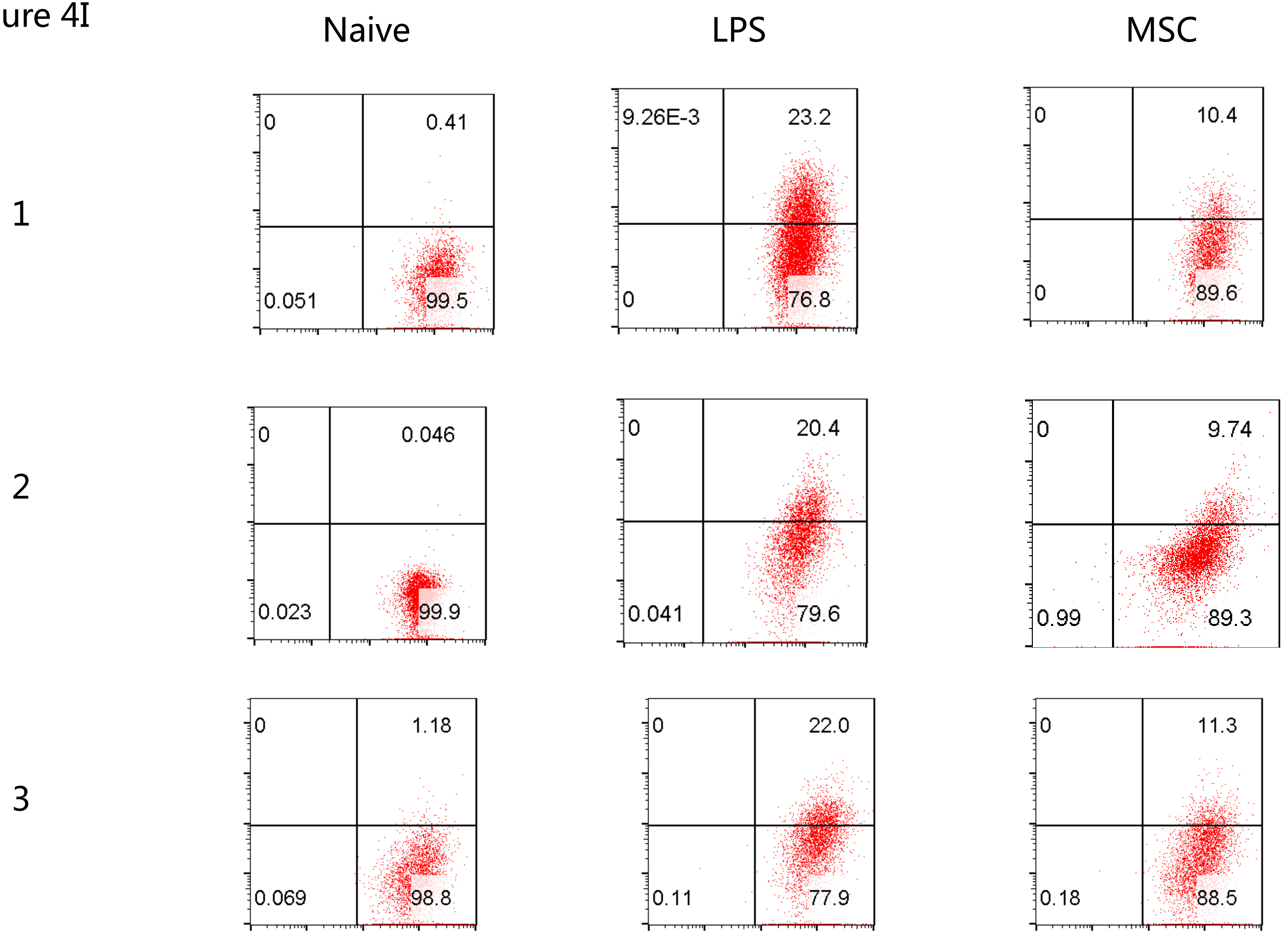
Naive
LPS
MSC
1
2
3
0
0.41
0.051
99.5
9.26E-3
23.2
0
76.8
0
10.4
0
89.6
0
0.046
0.023
99.9
0
20.4
0.041
79.6
0
9.74
0.99
89.3
0
1.18
0.069
98.8
0
22.0
0.11
77.9
0
11.3
0.18
88.5

Figure 5G

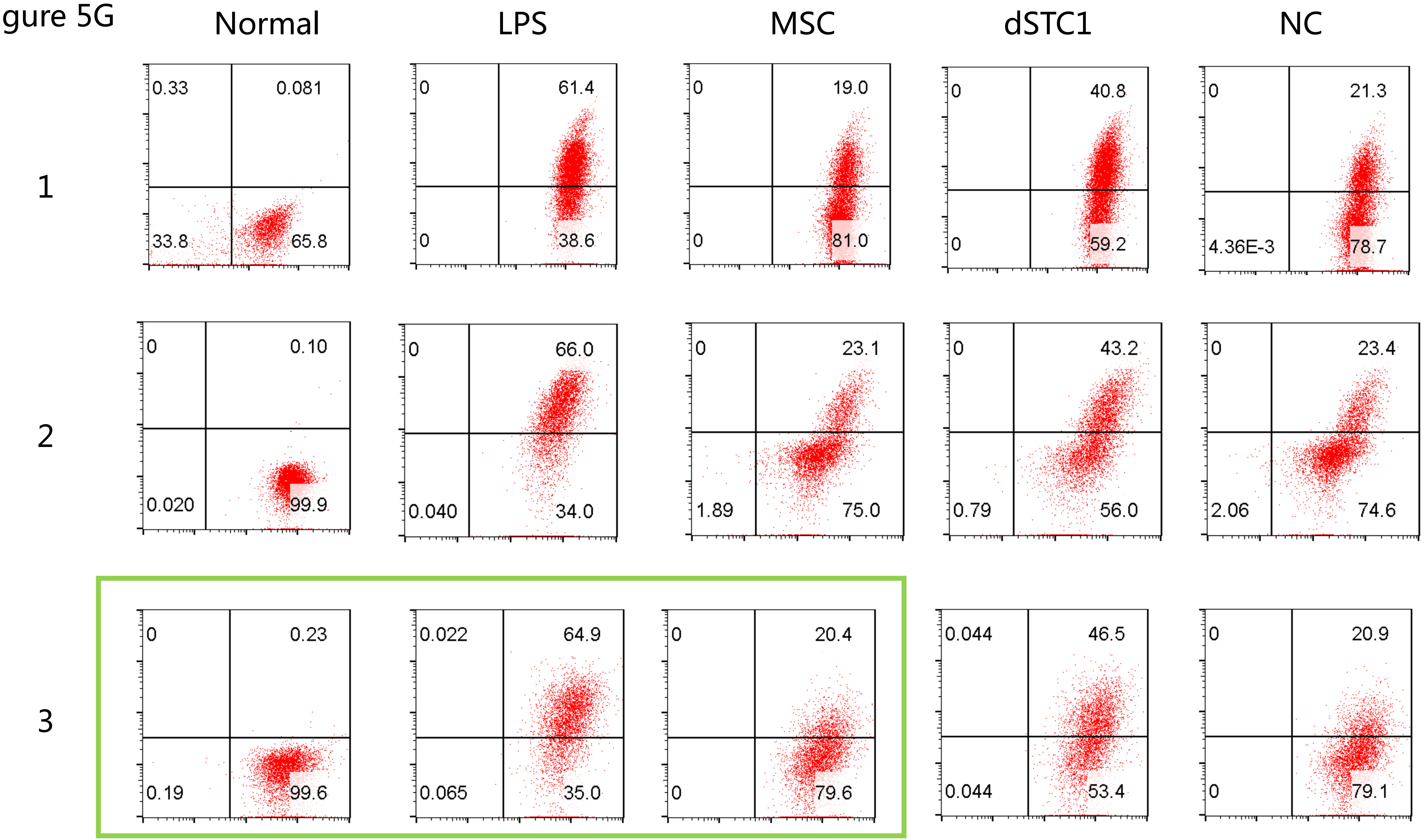
Normal
LPS
MSC
dSTC1
NC
1
0.33
0.081
33.8
65.8
0
61.4
0
38.6
0
19.0
0
81.0
0
40.8
0
59.2
0
21.3
4.36E-3
78.7
2
0
0.10
0.020
99.9
0
66.0
0.040
34.0
0
23.1
1.89
75.0
0
43.2
0.79
56.0
0
23.4
2.06
74.6
3
0
0.23
0.19
99.6
0.022
64.9
0.065
35.0
0
20.4
0
79.6
0.044
46.5
0.044
53.4
0
20.9
0
79.1

Figure 5J

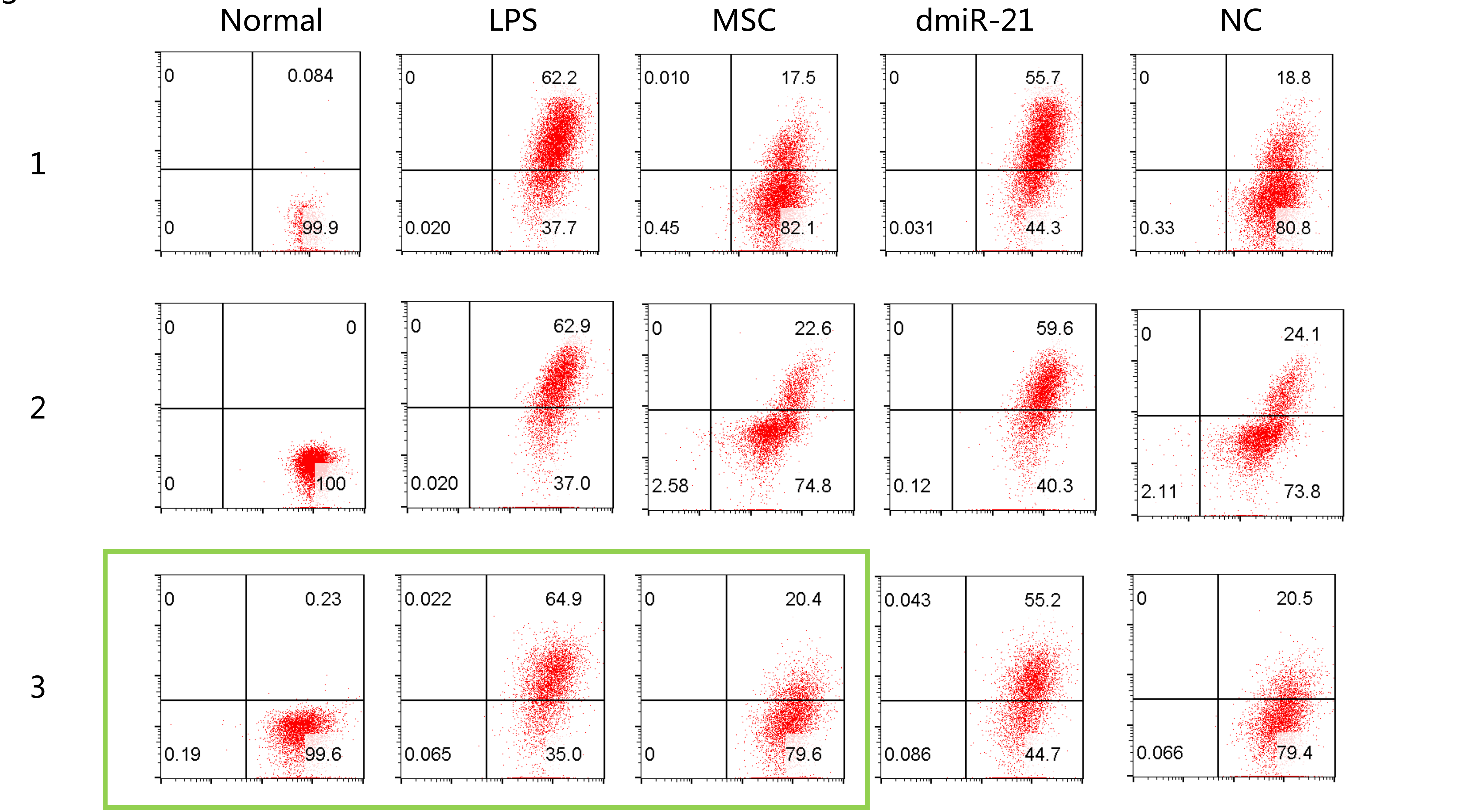

Normal
LPS
MSC
dmiR-21
NC
1
2
3
0
0.084
0
99.9
0
62.2
0.020
37.7
0.010
17.5
0.45
82.1
0
55.7
0.031
44.3
0
18.8
0.33
80.8
0
0
0
100
0
62.9
0.020
37.0
0
22.6
2.58
74.8
0
59.6
0.12
40.3
0
24.1
2.11
73.8
0
0.23
0.19
99.6
0.022
64.9
0.065
35.0
0
20.4
0
79.6
0.043
55.2
0.086
44.7
0
20.5
0.066
79.4

Note:
Data of Figure 5G #3 and Figure 5J #3 were from the same experiment. Thus, the Normal, LPS, and MSC groups are the same (indicated by green box).

Figure 5M

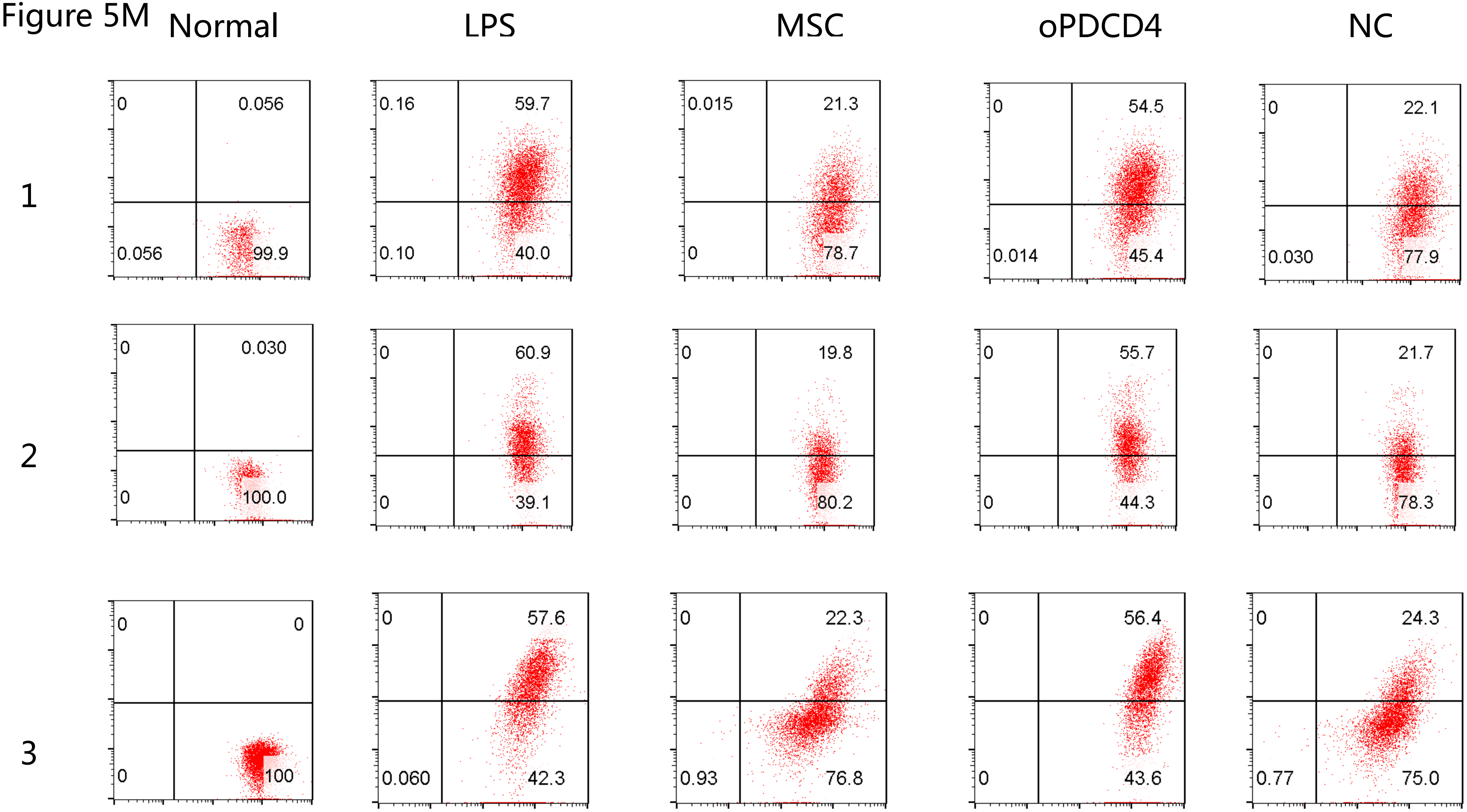

Normal
LPS
MSC
oPDCD4
NC
1
0
0.056
0.056
99.9
0.16
59.7
0.10
40.0
0.015
21.3
0
78.7
0
54.5
0.014
45.4
0
22.1
0.030
77.9
2
0
0.030
0
100.0
0
60.9
0
39.1
0
19.8
0
80.2
0
55.7
0
44.3
0
21.7
0
78.3
3
0
0
0
100
0
57.6
0.060
42.3
0
22.3
0.93
76.8
0
56.4
0
43.6
0
24.3
0.77
75.0

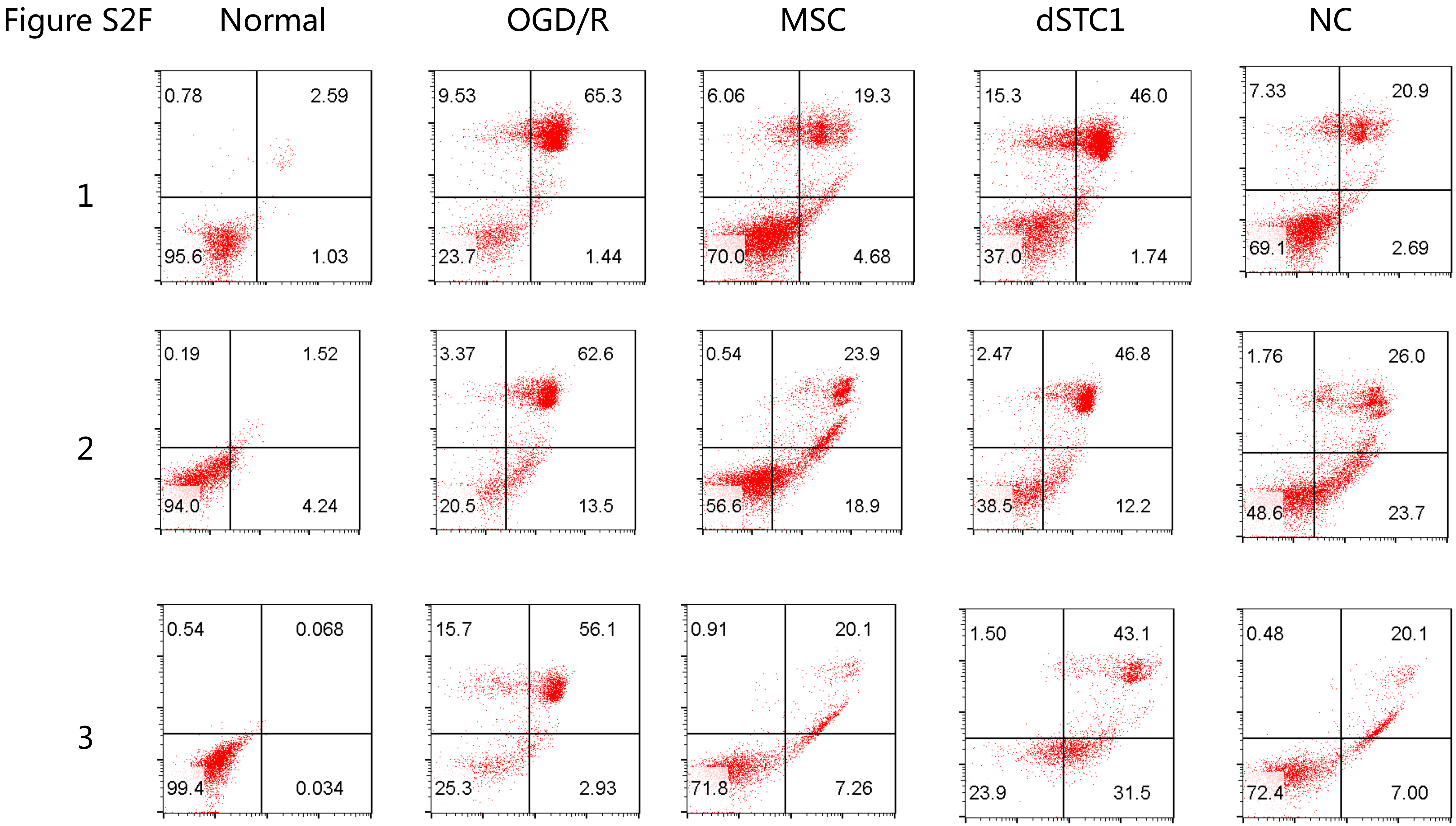
Figure S2F
Normal
OGD/R
MSC
dSTC1
NC
1
2
3
0.78
2.59
95.6
1.03
9.53
65.3
23.7
1.44
6.06
19.3
70.0
4.68
15.3
46.0
37.0
1.74
7.33
20.9
69.1
2.69
0.19
1.52
94.0
4.24
3.37
62.6
20.5
13.5
0.54
23.9
56.6
18.9
2.47
46.8
38.5
12.2
1.76
26.0
48.6
23.7
0.54
0.068
99.4
0.034
15.7
56.1
25.3
2.93
0.91
20.1
71.8
7.26
1.50
43.1
23.9
31.5
0.48
20.1
72.4
7.00

Figure S3B

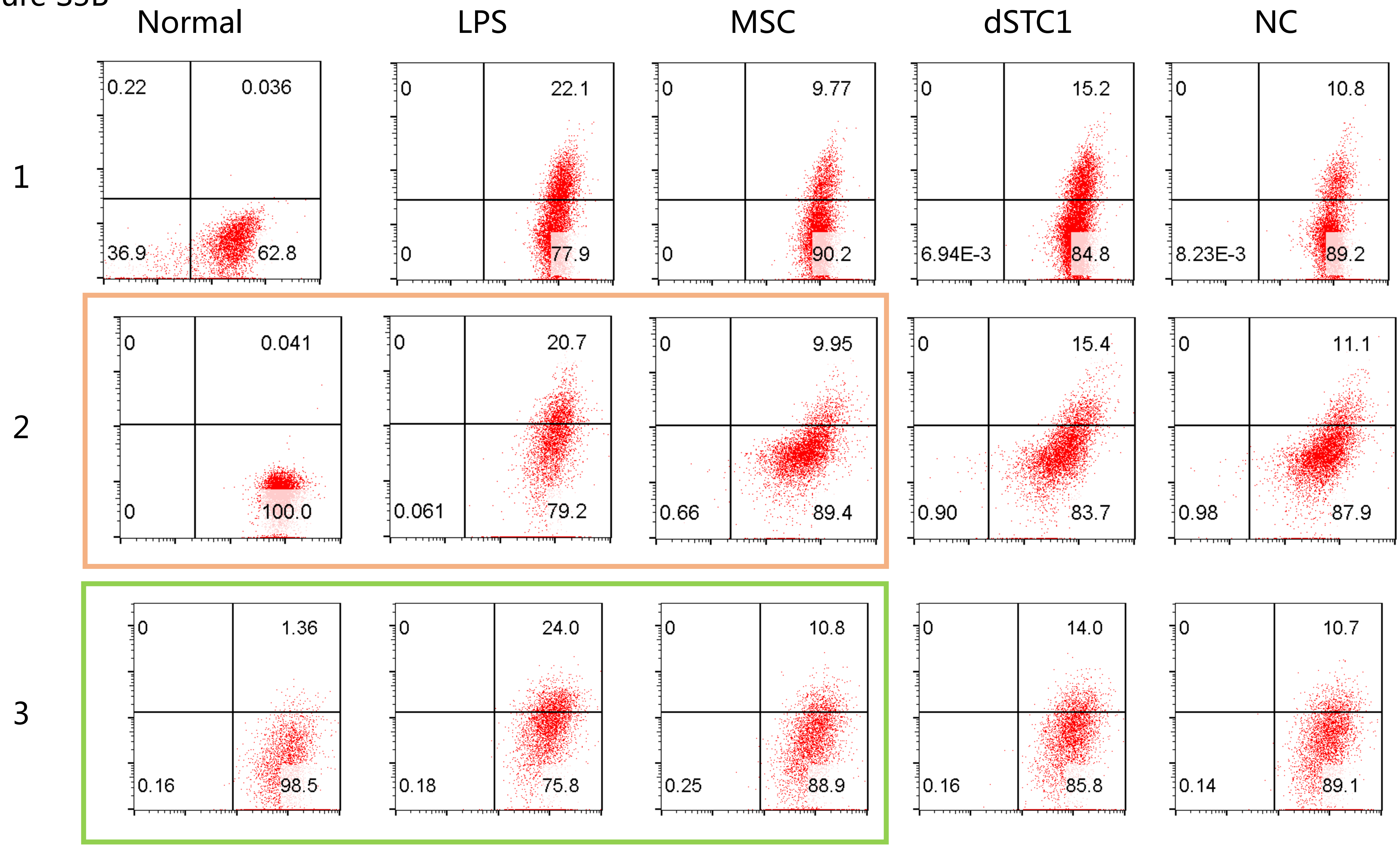
Normal
LPS
MSC
dSTC1
NC
1
2
3
0.22
0.036
36.9
62.8
0
22.1
0
77.9
0
9.77
0
90.2
0
15.2
6.94E-3
84.8
0
10.8
8.23E-3
89.2
0
0.041
0
100.0
0
20.7
0.061
79.2
0
9.95
0.66
89.4
0
15.4
0.90
83.7
0
11.1
0.98
87.9
0
1.36
0.16
98.5
0
24.0
0.18
75.8
0
10.8
0.25
88.9
0
14.0
0.16
85.8
0
10.7
0.14
89.1

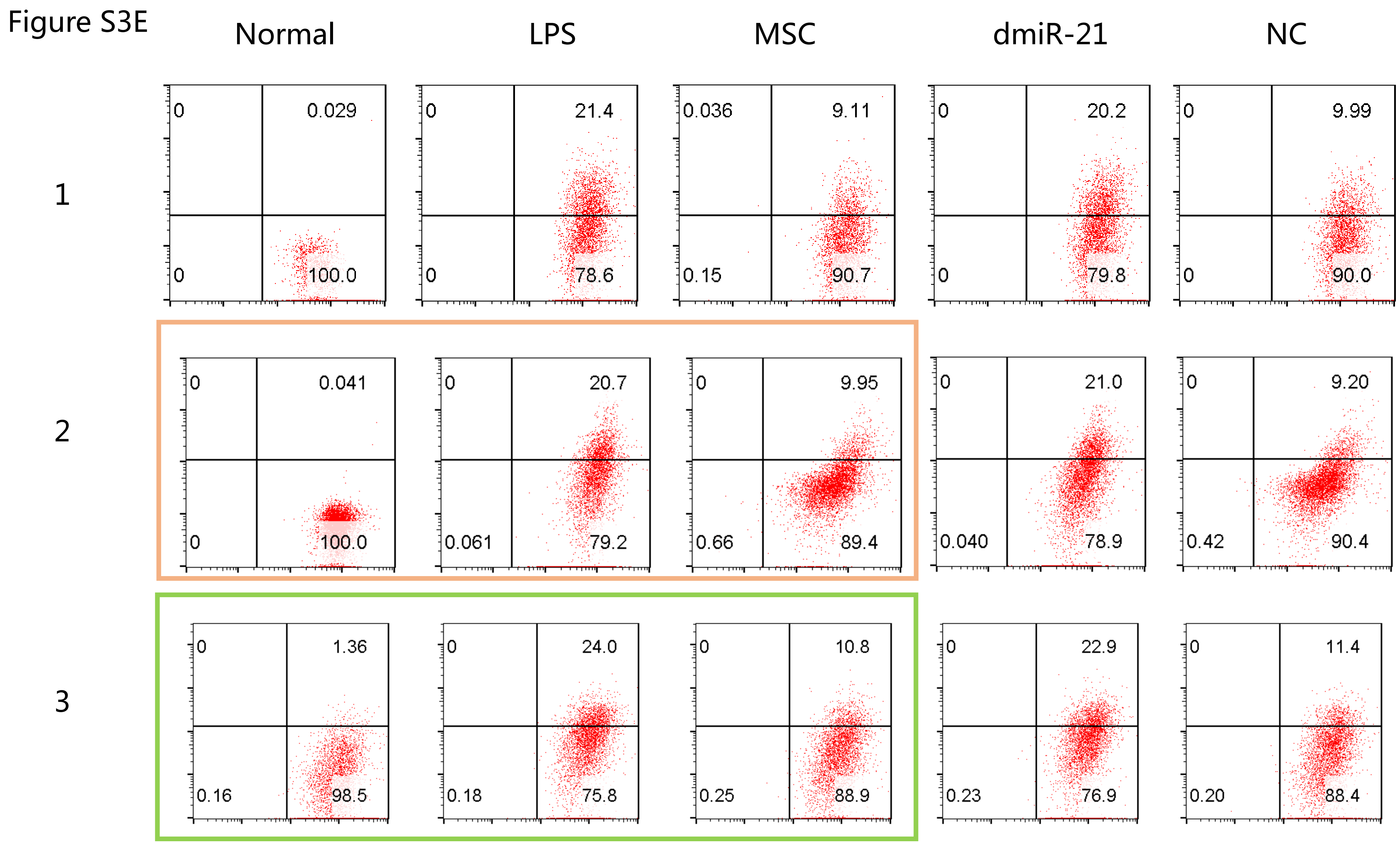
Figure S3E
Normal
LPS
MSC
dmiR-21
NC
1
2
3
0
0.029
0
100.0
0
21.4
0
78.6
0.036
9.11
0.15
90.7
0
20.2
0
79.8
0
9.99
0
90.0
0
0.041
0
100.0
0
20.7
0.061
79.2
0
9.95
0.66
89.4
0
21.0
0.040
78.9
0
9.20
0.42
90.4
0
1.36
0.16
98.5
0
24.0
0.18
75.8
0
10.8
0.25
88.9
0
22.9
0.23
76.9
0
11.4
0.20
88.4

Figure S3H

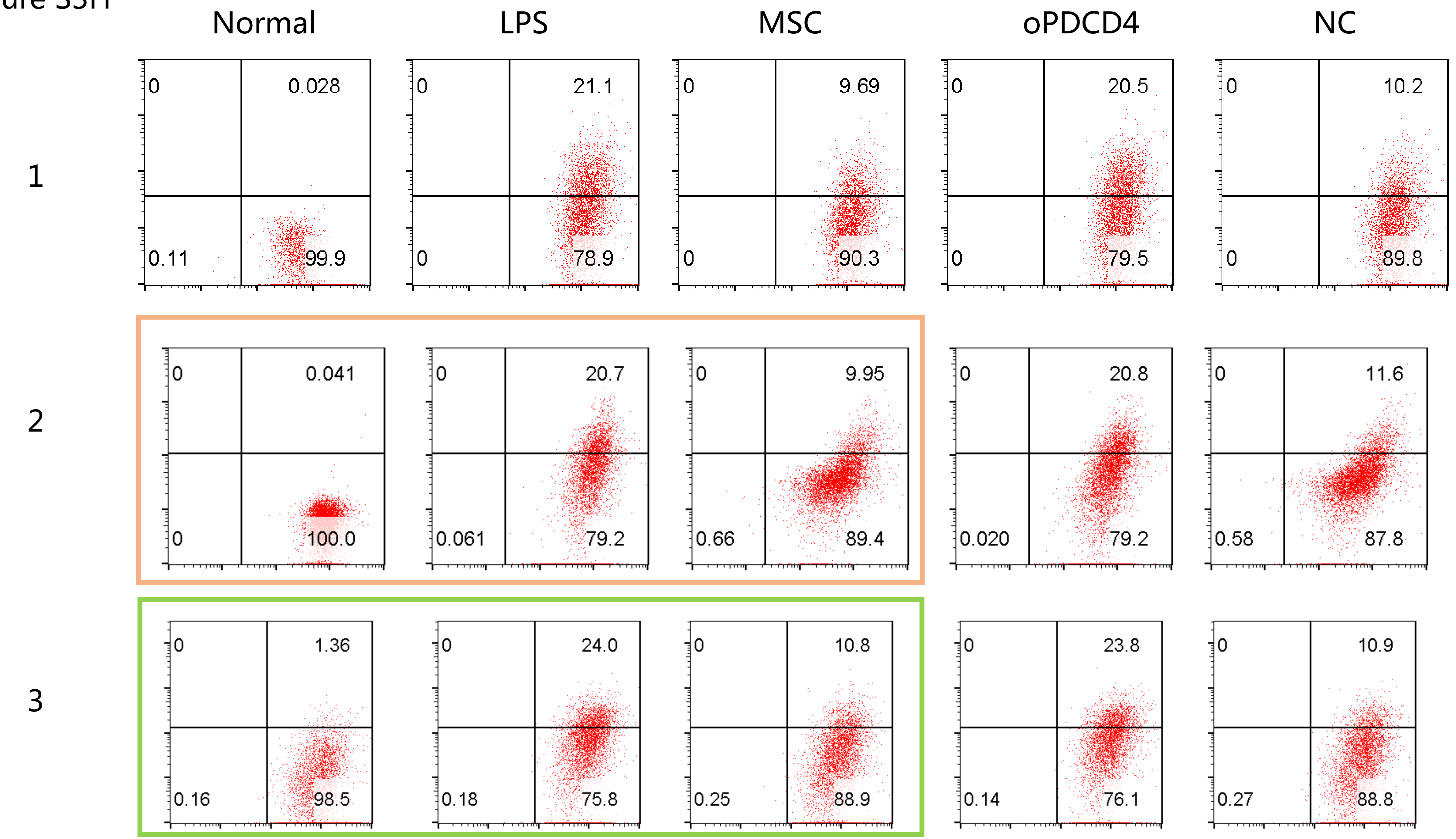

Normal
LPS
MSC
oPDCD4
NC
1
2
3
0
0.028
0.11
99.9
0
21.1
0
78.9
0
9.69
0
90.3
0
20.5
0
79.5
0
10.2
0
89.8
0
0.041
0
100.0
0
20.7
0.061
79.2
0
9.95
0.66
89.4
0
20.8
0.020
79.2
0
11.6
0.58
87.8
0
1.36
0.16
98.5
0
24.0
0.18
75.8
0
10.8
0.25
88.9
0
23.8
0.14
76.1
0
10.9
0.27
88.8

Note:

Data of Figure S3B #2, Figure S3E #2, and Figure S3H #2 were from the same experiment.

Data of Figure S3B #3, Figure S3E #3, and Figure S3H #3 were from the same experiment.

Thus, the Normal, LPS, and MSC groups are the same (indicated by orange and green boxes, respectively).
